# Metformin restores crizotinib sensitivity in crizotinib-resistant human lung cancer cells through inhibition of IGF1-R signaling pathway

**DOI:** 10.18632/oncotarget.9120

**Published:** 2016-04-30

**Authors:** Li Li, Yubo Wang, Tao Peng, Kejun Zhang, Caiyu Lin, Rui Han, Conghua Lu, Yong He

**Affiliations:** ^1^ Department of Respiratory Disease, Daping Hospital, Third Military Medical University, Chongqing 400042, China; ^2^ Department of Clinical Labratory, Daping Hospital, Third Military Medical University, Chongqing 400042, China

**Keywords:** metformin, crizotinib, IGF-1R, lung cancer, resistance

## Abstract

**Aim:**

Despite the impressive efficacy of crizotinib for the treatment of ALK-positive non-small cell lung cancer, patients invariably develop therapeutic resistance. Suppression of the IGF-1R signaling pathway may abrogate this acquired mechanism of drug resistance to crizotinib. Metformin, a widely used antidiabetic agent, may reverse crizotinib resistance through inhibition of IGF-1R signaling.

**Results:**

The present study revealed that metformin effectively increased the sensitivity of both crizotinib-sensitive and -resistant non-small cell lung cancer cells to crizotinib, as evidenced by decreased proliferation and invasion and enhanced apoptosis. Metformin reduced IGF-1R signaling activation in crizotinib-resistant cells. Furthermore, the addition of IGF-1 to crizotinib-sensitive H2228 cells induced crizotinib resistance, which was overcome by metformin.

**Experimental design:**

The effects of metformin to reverse crizotinib resistance were examined *in vitro* by using 3-(4,5-dimethylthiazol-2-yl)-2,5-diphenyltetrazolium (MTT), invasion assay, ki67 incorporation assay, flow cytometry analysis, Western blot analysis, and colony-forming assay.

**Conclusions:**

Metformin may be used in combination with crizotinib in ALK+ NSCLC patients to overcome crizotinib resistance and prolong survival.

## INTRODUCTION

Genomic alterations in the anaplastic lymphoma kinase (ALK) gene are found in numerous malignancies [1]. ALK rearrangements, first discovered by Soda et al. in 2007 [2], are either inversions or translocations of the ALK-tyrosine kinase (-TK) receptor gene with other fusion partners. The expression of the echinoderm microtubule-associated protein-like 4 (EML4)-ALK fusion oncogene, which is caused by a small inversion within chromosome 2p, was found in non-small cell lung cancer (NSCLC) [2]. The product of the ALK rearrangement consists of a constitutively activated receptor TK with pro-oncogenic effects [3]. Consequently, tumors with ALK rearrangements are associated with ALK signaling, thus being effectively inhibited by small molecules ALK-TK inhibitors (-TKIs).

Crizotinib is a multitargeted TKI with activity against MET, ALK, and ROS1 [4, 5]. This active substance has been found to greatly outperform the best chemotherapy agents in NSCLC patients with ALK rearrangements [6, 7]. Recently, the PROFILE 1014 study demonstrated that crizotinib significantly improved PFS and ORR in the first-line treatment for ALK-positive NSCLC patients, with an acceptable safety profile, thus establishing crizotinib as a standard of care for previously untreated ALK-positive patients [8]. However, acquired resistance eventually develops in most patients. Two main mechanisms of crizotinib resistance have been reported. One of them is the alteration in the gene itself, including second-site ALK mutations and ALK copy number gain. The other mechanism is activation of alternative signaling pathways, including the development of EGFR mutations or activation of the wild-type EGFR, HER2-, or KIT-receptors [9–12]. Thus, innovative treatment strategies are urgently needed to overcome therapeutic resistance to crizotinib to improve the survival of NSCLC patients.

Molecular mechanisms underlying acquired crizotinib resistance are still not fully elucidated, mainly due to the small number of experimental results and included patients. Gatekeeper mutations, such as L1196M, L1152R, C1156Y, and F1174L hinder drug binding and are frequently detected in crizotinib-resistant samples [13, 14]. Besides, the IGF-1R signaling pathway is involved in crizotinib resistance. In a previous study, the addition of IGF-1 induced resistance to the inhibitory effects of crizotinib on growth. Chronic ALK inhibition was associated with enhanced IGF-1R signaling. Furthermore, the addition of an IGF-1R inhibitor sensitized the cells resistant to the effects of ALK blockade [15]. Thus, the IGF-1R signaling axis is a potential therapeutic target in ALK-positive non-small cell lung cancer.

Metformin is an insulin-sensitizing agent that has been used over 40 years to treat type II diabetes. Metformin has aroused keen interest as a potential anticancer agent ever since the report was published of the clinical evidence that the cancer risk and mortality were reduced in diabetics who received metformin [16]. Previously, we reported that metformin in combination with gefitinib or erlotinib had a synergistic inhibitory effect on the proliferation, migration, and invasion of cell lines resistant to EGFR-TKIs (epidermal growth factor receptor-tyrosine kinase inhibitors) [17]. However, it remains unknown whether metformin may overcome crizotinib resistance. Interestingly, metformin has been found to exert an inhibitory effect on IGF-1R signaling [18, 19], which encouraged us to perform the present study aimed at investigating whether metformin could restore crizotinib sensitivity in crizotinib-resistant cells through inhibition of the IGF-1R signaling pathway.

## RESULTS

### Metformin increased crizotinib sensitivity in crizotinib-sensitive human non-small cell lung cancer cells

**Figure 1:**
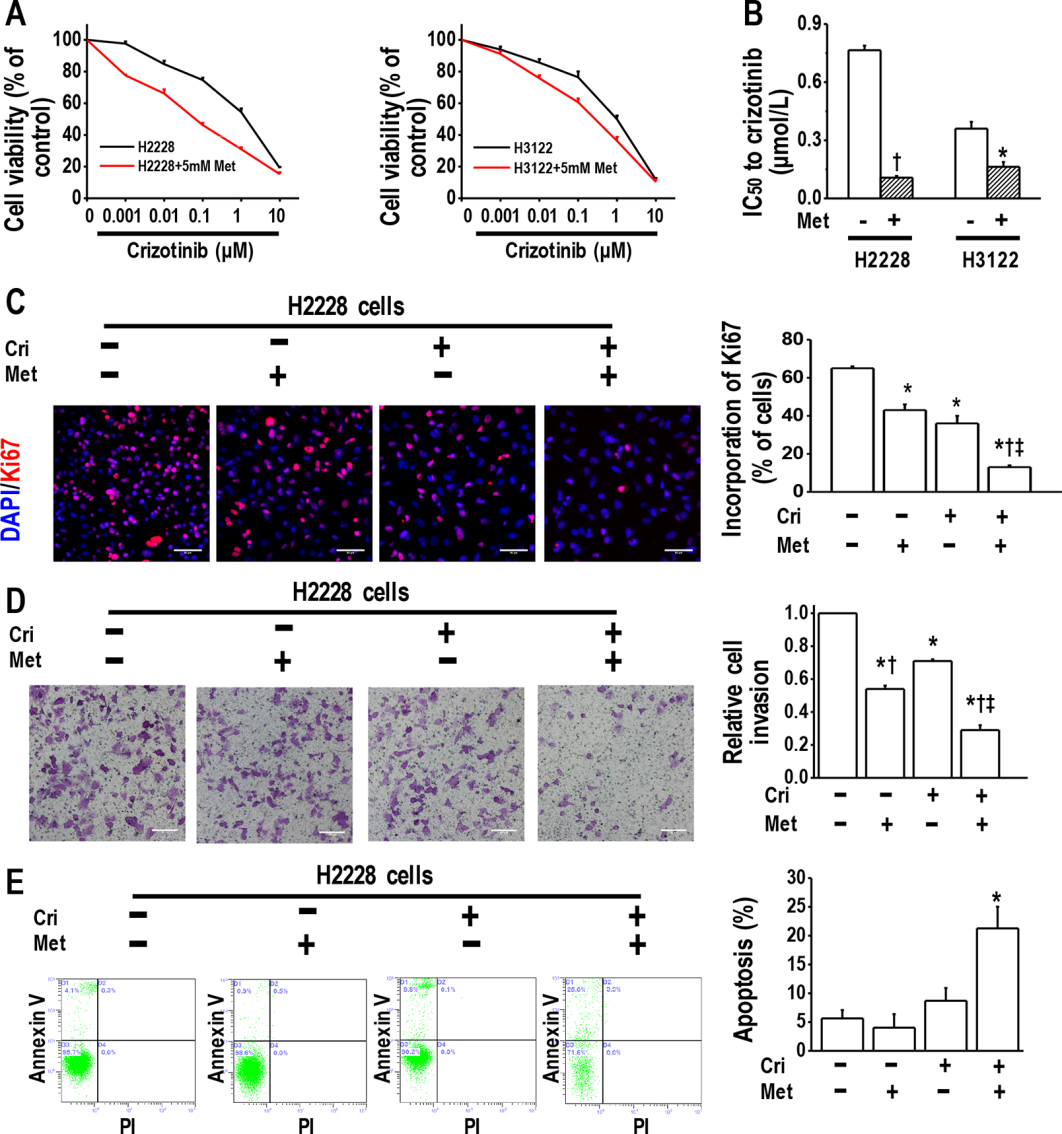
Metformin increased crizotinib sensitivity in crizotinib-sensitive human lung cancer cells (**A**) Metformin (5 mM) increased the sensitivity of H2228 and H3122 cells to crizotinib. The cell viability of H2228 and H3122 cells treated with the indicated doses of crizotinib for 48 h was assessed through the MTT method; (**B**) IC50 values to crizotinib of H2228 and H3122 cells, with or without metformin treatment *p < 0.05, ^†^*p* < 0.01 compared with that without metformin treatment; (**C**) Metformin (5 mM) and crizotinib (400 nM) synergistically inhibited the proliferation of H2228 cells, as determined by a Ki67 incorporation assay. ^*^p < 0.01 compared with control, ^†^*p* < 0.01 compared with that of crizotinib treatment alone, ^‡^*p* < 0.01 compared with that of metformin treatment alone. Scale bars, 50 μm; (**D**) Metformin (5 mM) and crizotinib (400 nM) synergistically inhibited invasiveness of H2228 cells. Scale bars: 100 μm. ^*^p < 0.01 compared with control; ^†^*p* < 0.01 compared with the crizotinib treatment alone; ^‡^*p* < 0.05 compared with that of metformin treatment alone; (**E**) Metformin (5 mM) in combination with crizotinib (400 nM) significantly enhanced the apoptosis of H2228 cells. The images are representative of three independent experiments. ^*^p < 0.01 compared with that of control, metformin treatment or crizotinib treatment. Met, metformin; Cri, crizotinib.

We first performed MTT assays to establish whether metformin could enhance the inhibitory action of crizotinib on the growth of crizotinib-sensitive H2228 and H3122 cells. H2228 and H3122 cells were sensitive to crizotinib, with IC50 values of 0.76 ± 0.02 μM and 0.36 ± 0.04 μM, respectively. The treatment with 5 mM metformin further increased the sensitivity of those cells to crizotinib (Figure 1A and 1B).

We next performed a Ki67 incorporation assay to confirm the effect of metformin in combination with crizotinib since metformin disrupts mitochondrial respiration, which may affect the MTT assay results. We revealed that the combination of metformin and crizotinib caused substantial inhibition of the cell proliferation of H2228 and H3122 cells (Figure 1C and Supplementary Figure 1).

Then, we performed a transwell assay to determine whether the drug combination exerted a more pronounced inhibitory effect on tumor cell invasion. It was found that metformin or crizotinib alone decreased the invasion ability of H2228 and H3122 cells, whereas the combination of metformin and crizotinib further enhanced this effect (Figure 1D and Supplementary Figure 1).

We next analyzed the induction of apoptosis in H2228 cells treated with metformin alone or in combination with crizotinib. The flow cytometry analysis results revealed that metformin in combination with crizotinib significantly enhanced the apoptosis of H2228 cells (Figure 1E). The same finding was observed in H3122 cells treated with metformin, or crizotinib, or both (Supplementary Figure 1). Of note, metformin of 5 mM only slightly decreased cell viability in cells used in the current study (Supplementary Figure 2). These *in vitro* data suggest that when applied in combination, metformin increases crizotinib sensitivity in crizotinib-sensitive cells.

### Metformin reversed crizotinib resistance in crizotinib-resistant cells

**Figure 2:**
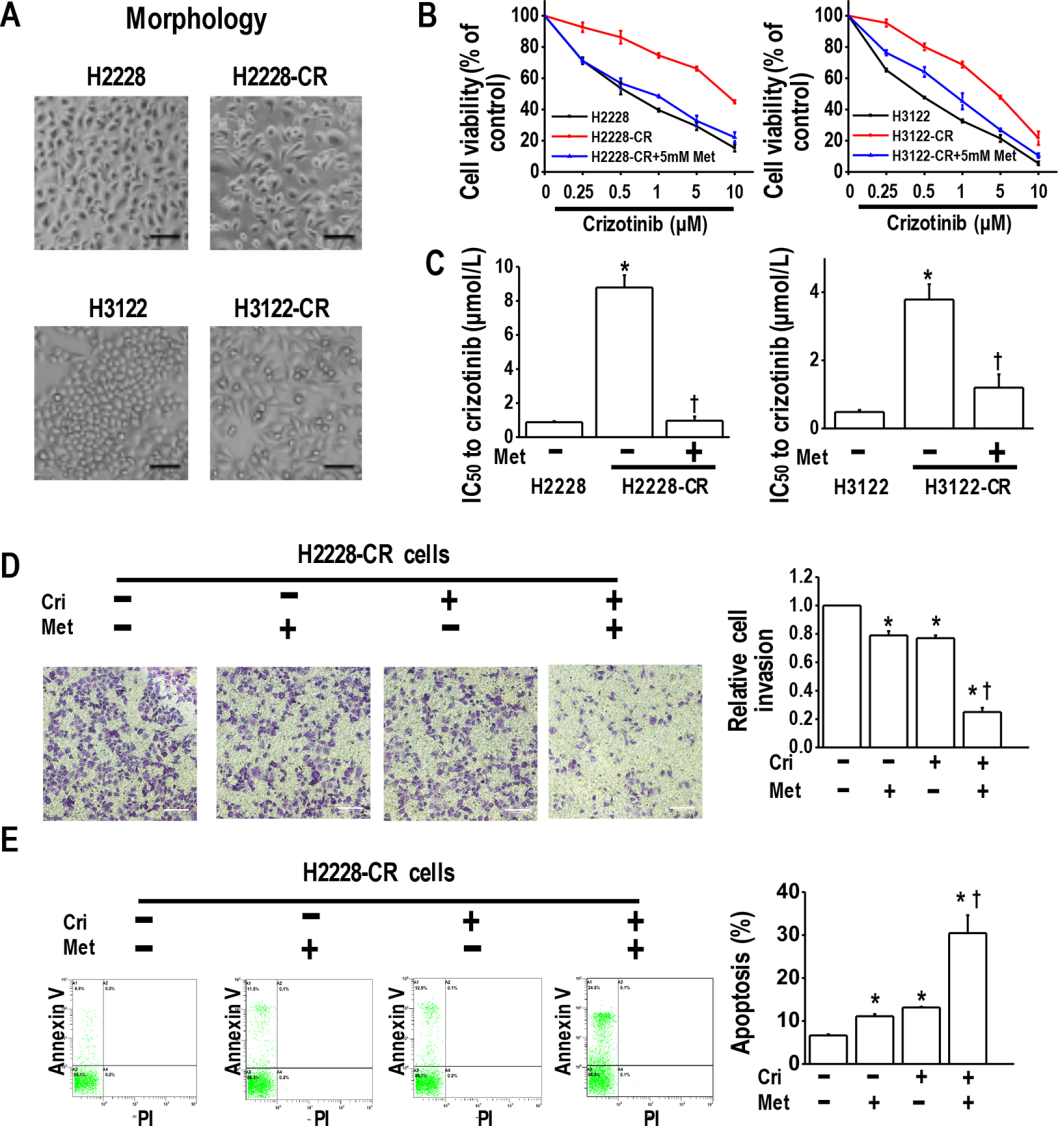
Metformin resensitized crizotinib-resistant human lung cancer cells to crizotinib (**A**) Morphology of parental cells and crizotinib-resistant cells; (**B**) Metformin (5 mM) increased the sensitivity of H2228-CR cells and H3122-CR cells to crizotinib. Parental cells and crizotinib resistant cells were treated with the indicated doses of crizotinib for 48 h. The cell viability, assessed by the MTT method, was expressed as % of control for each time point; (**C**) IC50 values to crizotinib of H2228-CR cells and H3122-CR cells, with or without metformin treatment *p < 0.01 compared with parental cells; ^†^*p* < 0.01 compared with the resistant cells without metformin treatment; (**D**) Metformin and crizotinib synergistically inhibited invasiveness of H2228-CR cells. Metformin- and/or crizotinib-treated H2228-CR cells were seeded into transwell plates for 48 h, and then the invasive cells were fixed and stained with crystal violet. Scale bars: 100 μm. *p < 0.01 compared with that of the control; ^†^*p* < 0.01 compared with that of metformin or crizotinib treatment alone; (**E**) Metformin (5 mM) in combination with crizotinib (5 μM) significantly enhanced the apoptosis of H2228 cells. The images are representative of three independent experiments. *p < 0.05 compared with that of the control, ^†^*p* < 0.05 compared with the metformin or crizotinib treatment. Met, metformin; Cri, crizotinib.

We next speculated whether metformin could overcome crizotinib resistance in crizotinib-resistant human lung cancer cells. For this purpose, we established two crizotinib-resistant sublines (H2228-CR and H3122-CR cells), which were derived from the parental H2228 and H3122 cell lines by long-term exposure to high concentrations of crizotinib for eight months. Typical epithelial morphology features were observed in H2228 and H3122 cells, whereas spindle-cell shapes were observed in H2228-CR and H3122-CR cells (Figure 2A). Further, the MTT results indicated that H2228-CR cells and H3122-CR cells exhibited higher resistance to crizotinib than the parental cell lines, while the addition of metformin significantly increased the sensitivity of both resistant cell lines to crizotinib (Figure 2B and 2C).

By transwell assay, we investigated whether metformin could decrease tumor invasion in crizotinib resistant H2228-CR and H3122-CRcells. The results showed that metformin or crizotinib alone decreased slightly the cell invasion, whereas the drug combination significantly enhanced this inhibitory effect (Figure 2D and Supplementary Figure 3). Next, by using flow cytometry analysis, we found that metformin utilized together with crizotinib had a synergistic effect of enhancing the apoptosis of H2228-CR cells and H3122-CR cells (Figure 2E and Supplementary Figure 3). Taken together, these data suggest that metformin resensitized the cells resistant to crizotinib by inhibiting their proliferation and invasion and promoting apoptosis.

### Metformin decreased IGF1-R signaling in both crizotinib-sensitive and -resistant cell lines

**Figure 3:**
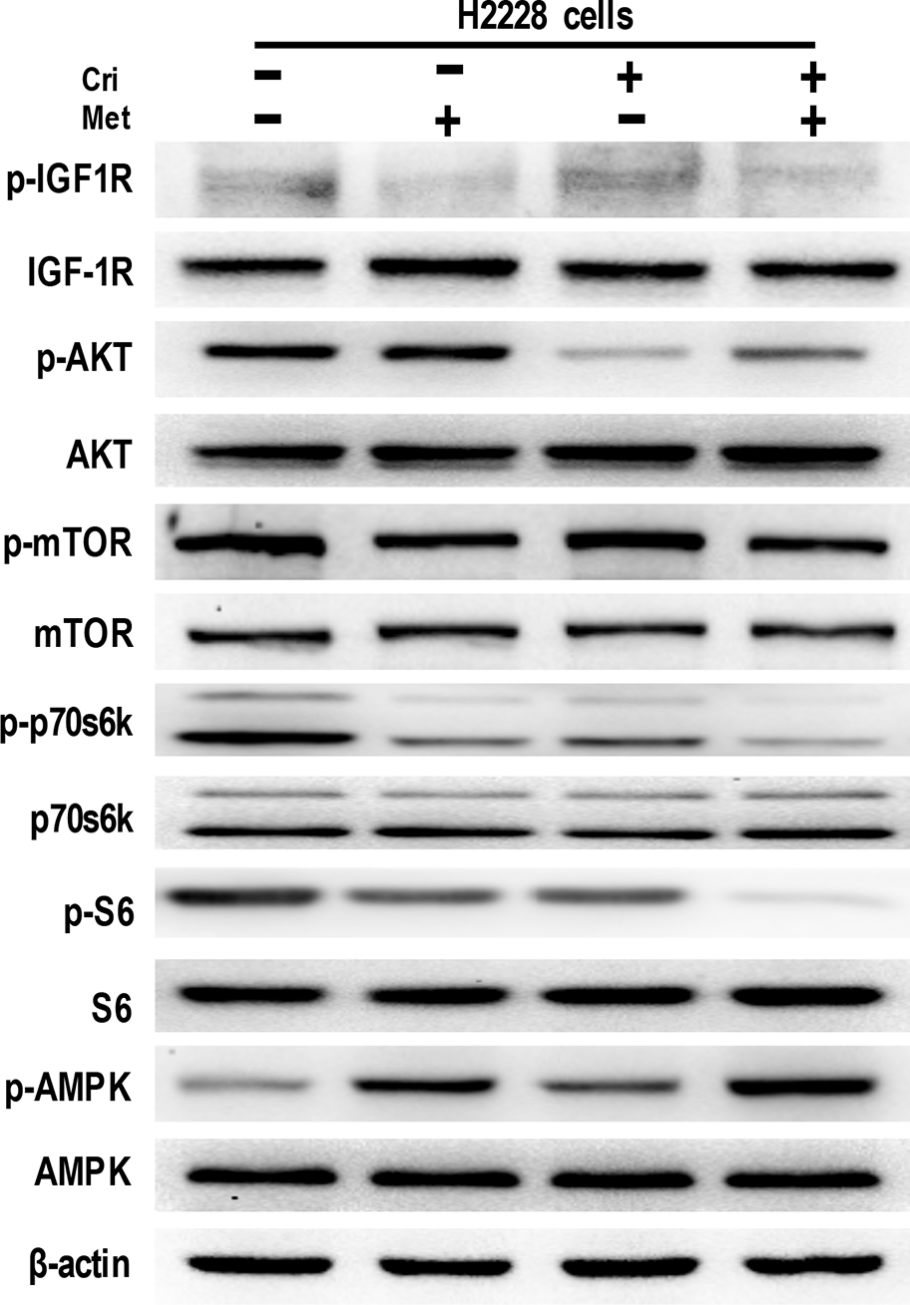
Metformin decreased IGF-1R signaling in crizotinib-sensitive human lung cancer cells Whole cell protein lysates from H2228 cells with different treatments were immunoblotted with antibodies as indicated, and β-actin was used to confirm the equal gel loading. Similar results were obtained in three independent experiments.

To identify the molecular mechanisms of metformin in overcoming acquired crizotinib resistance, we examined the effect of metformin on IGF1-R signaling activation, which was reported to be the key mechanism for crizotinib resistance [15]. Starting with drug sensitive H2228 cells, we performed a Western Blot analysis and found that metformin alone or in combination with crizotinib decreased IGF-1R phosphorylation, whereas the separate use of crizotinib enhanced IGF-1R phosphorylation. Concerning the effect exerted on the downstream signaling molecules, we discovered that metformin alone or in combination with crizotinib reduced the phosphorylation levels of mTOR, p70s6k, and S6, whereas it did not alter the activation of AKT (Figure 3).

Further, we investigated the possible mechanism by which metformin reduced IGF-1R signaling activation. It was reported that metformin disrupted mitochondrial respiration, which then increased the intracellular ratio of AMP: ATP and induced activation of AMPK [20]. We thus analyzed the activation of AMPK caused by the metformin treatment. The results showed that metformin alone or in combination significantly stimulated the activation of AMPK (Figure 3). Taken together, these data suggest that metformin decreases IGF-1R signaling activation in H2228 cells.

**Figure 4:**
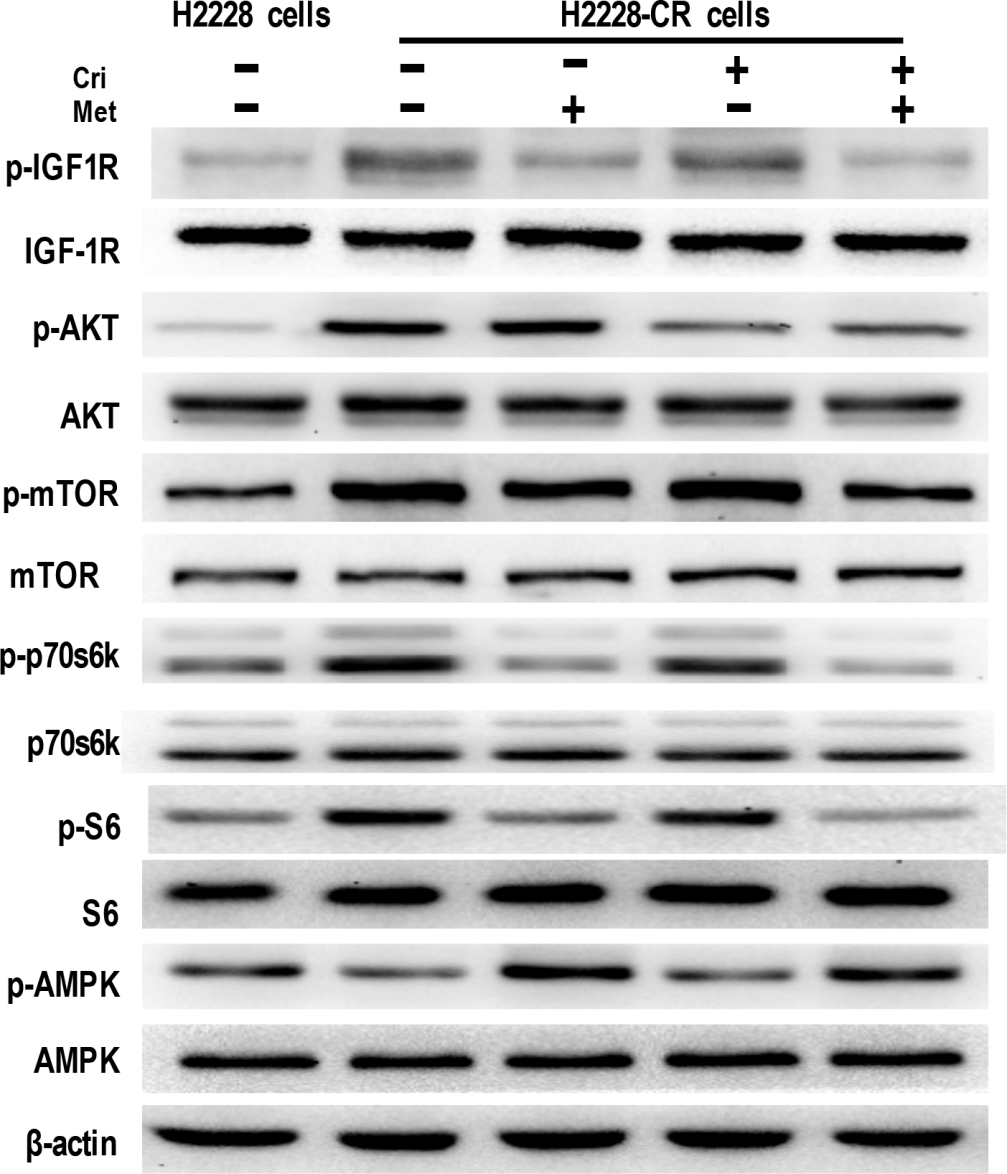
Metformin decreased IGF-1R signaling in crizotinib-resistant human lung cancer cells The expression of the indicated markers on protein extracts obtained from H2228 cells and H2228-CR cells with different treatments was analyzed via Western blotting, and β-actin was used as a loading control. Similar results were obtained in three independent experiments.

We next attempted to elucidate whether IGF-1R signaling manifested enhanced activation in H2228-CR cells, and whether metformin could attenuate it. The results from the Western Blot analysis showed that IGF-1R phosphorylation was elevated in the ALK-TKI-resistant cells, together with upregulated expression of phosphorylated proteins, including AKT, mTOR, p70s6k, and S6. Similarly to its effect in H2228 cells, metformin alone or in combination with crizotinib decreased the expression of phosphorylated IGF-1R, mTOR, p70s6k, and S6. Also, decreased activation of AMPK was noted in H2228-CR cells when compared to that of the parental cells, whereas metformin promoted the phosphorylation of AMPK in the resistant cells (Figure 4). Overall, these data showed that metformin suppressed the IGF-1R/mTOR/S6 signaling pathway in both crizotinib-sensitive and -resistant H2228-CR cells.

### Metformin reversed IGF-1-induced crizotinib resistance

**Figure 5:**
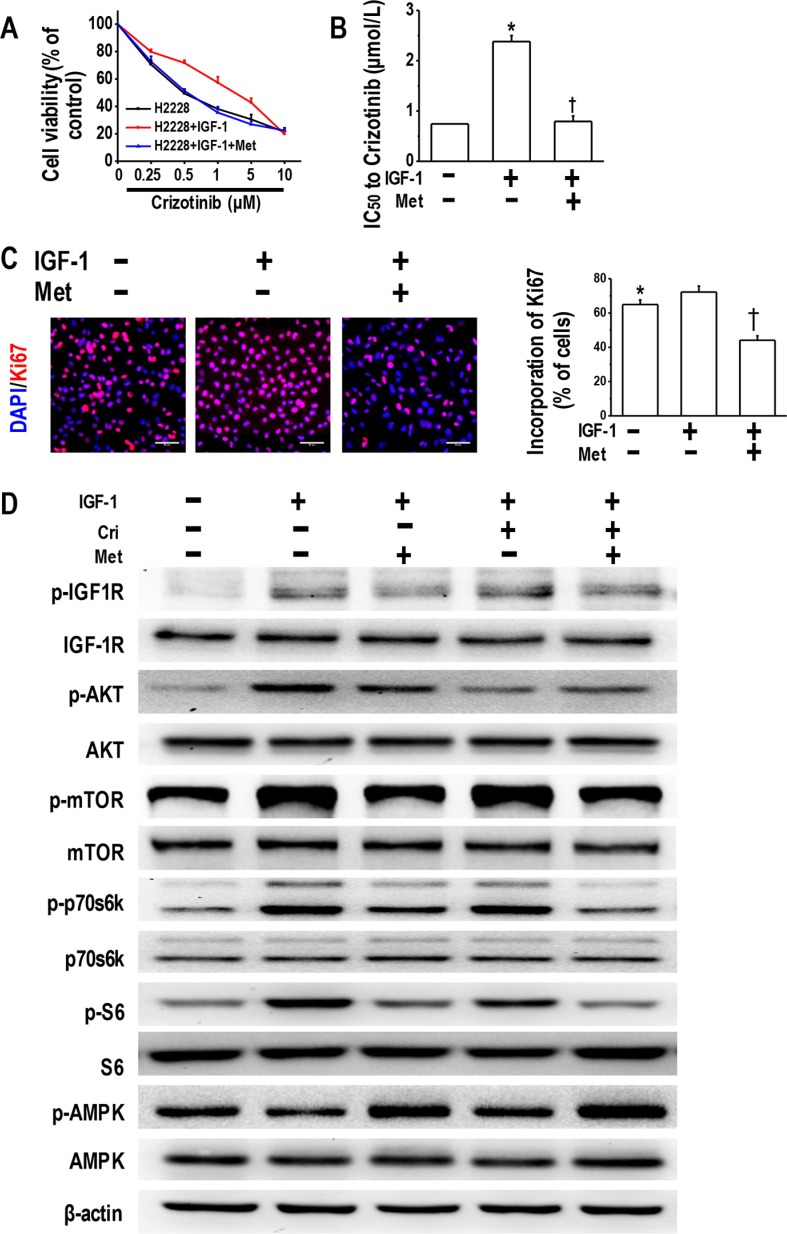
Metformin reversed IGF-1-induced crizotinib resistance and decreased IGF-1R signaling activation (**A**) Metformin reversed IGF-1-induced crizotinib-resistance in the parental H2228 cells. Parental H2228 cells (untreated, or treated with 100 ng/mL IGF-1 or IGF-1 plus 5 mM metformin) were incubated with crizotinib at the indicated concentrations. The cell viability was assessed via the MTT method after 48-h treatment; (**B**) IC50 values to crizotinib of H2228 cells of different treatment *p < 0.01 compared with no treatment; ^†^*p* < 0.01 compared with the IGF-1 treatment alone; (**C**) Metformin (5 mM) inhibited the IGF-1-induced proliferation of H2228 cells, as determined by the Ki67 incorporation assay. The cells were counter-stained with 4′, 6-diamidino-2-phenylindole (DAPI). *p < 0.05 compared with the IGF-1 treatment alone, ^†^*p* < 0.01 compared with the IGF-1 treatment alone. Scale bars, 50 μm; (**D**) Metformin decreased the IGF-1R signaling activation in the IGF-1-stimulated parental H2228 cells. Whole cell protein lysates from H2228 cells with different treatments were immunoblotted with antibodies as indicated, and β-actin was used to confirm the equal gel loading. Similar results were obtained in three independent experiments.

Given that IGF-1 addition induced resistance to crizotinib [15], we next investigated whether metformin could overcome IGF-1-induced crizotinib resistance in H2228 cells. As illustrated in Figure 5A and 5B, 48-h culture in IGF-1-containing medium decreased the sensitivity of H2228 cells to crizotinib, whereas the metformin addition restored the sensitivity. We also performed Ki67 incorporation assay, and the results revealed that the addition of IGF-1 slightly enhanced cell proliferation (not significantly different), while the addition of metformin caused a decline in the incorporation of Ki67 (Figure 5C).

Next, we examined IGF-1R activation in H2228 cells treated with IGF-1 or IGF-1 plus metformin. The IGF-1 exposure resulted in significant phosphorylation of its downstream molecules, including IGF-1R, AKT, mTOR, p70s6k, and S6 and led to the decreased activation of AMPK. Metformin reduced the phosphorylation of IGF-1R, mTOR, p70s6k, and S6, and stimulated the activation of AMPK (Figure 5D). In summary, our data indicate that the IGF-1 activation of IGF-1R/mTOR/S6 pathway was sufficient to induce increased crizotinib resistance, whereas metformin successfully restored crizotinib sensitivity and decreased IGF-1R/mTOR/S6 signaling activation.

## DISCUSSION

In the present study, we report for the first time that metformin in combination with crizotinib not only enhances crizotinib sensitivity in crizotinib-sensitive cell lines, but reverses crizotinib resistance in crizotinib-resistant cell lines. The effect of metformin against crizotinib resistance was attributed to its ability to decrease IGF-1R signaling activation. We thus provide the rationale and experimental evidence for the combined use of metformin and crizotinib to overcome crizotinib resistance in ALK+ NSCLC patients.

### Clinical significance: Metformin overcomes crizotinib resistance

Patients with ALK gene rearrangements often manifest dramatic responses to crizotinib, while acquired drug resistance develops eventually and inevitably. One interesting approach to reversing crizotinib resistance would be that of adding another drug to crizotinib. A combination of crizotinib with pemetrexed, or a targeted agent, such as an Hsp90 inhibitor, is currently being tested in clinical trials in ALK-positive advanced NSCLCs [7]. In the present study, we also adopted this approach, by using metformin in combination with crizotinib. As expected, the addition of metformin enhanced the sensitivity of H2228 and H3122 cells to crizotinib. Moreover, in the crizotinib-resistant H2228-CR and H3122-CR cells, the combination of metformin reversed drug resistance, as shown by both MTT and Ki67 incorporation assays.

Metformin is an oral antidiabetic drug and is of emerging interest due to its anti-cancer capacity. In recent years, a number of retrospective epidemiological and pre-clinical studies have supported the use of metformin as an adjuvant in the chemotherapy for cancer treatment [21]. In lung cancer patients with diabetes, meta-analysis showed that the use of metformin improved survival [22]. A retrospective study from our group showed that in advanced NSCLC patients with type 2 diabetes, metformin together with EGFR-TKI resulted in longer PFS and OS [23]. Metformin can target cancer stem cells, which are hypothesized to be critical initiators of cancers [24]. In HER2-positive carcinomas, metformin in combination with the anti-HER2 monoclonal antibody trastuzumab could synergistically suppress the self-renewal and proliferation of cancer stem/progenitor cells [25]. Metformin is also involved in the targeted therapy of NSCLCs. Previously, we and other researchers reported that metformin in combination with EGFR-TKIs erlotinib or gefitinib had a synergistic inhibitory effect on the proliferation, migration, and invasion of cell lines resistant to TKIs [17, 26]. Taken together, data from the current study suggest that metformin, in combination with crizotinib, has the promising potential of turning into a novel anti-cancer agent that enhances the effect of crizotinib.

### IGF-1R signaling: the key for metformin to overcome crizotinib resistance

Understanding the molecular mechanisms by which metformin overcomes crizotinib resistance is essential to its development as a novel agent for the treatment of NSCLC patients with ALK rearrangement. Reportedly, IGF-1 signaling pathway is associated with crizotinib resistance. The levels of IGF-1 appear to correlate with cancer risk in human populations [27], and the high contents of IGF-1 are associated with increased mortality in the general population [28]. Elevated IGF-1 concentrations were observed in patients who had taken crizotinib [29]. The amounts of IGF-1R were greater in tumor biopsies taken at the time of acquired resistance when compared to those obtained from the respective pre-resistant tumors. Also, the combination of crizotinib and an IGF-1R inhibitor might be able to overcome this resistance [15]. The downstream signaling pathway, Akt/mTOR/S6 kinase pathway, was also associated with crizotinib resistance [30]. The activation of the mTOR pathway was related to increased autophagy of the ALK receptor, leading to a weakened response to crizotinib treatment [31]. A synergistic *in vitro* growth inhibitory effect was established when an ALK inhibitor was combined with an mTOR inhibitor [32]. Thus, we investigated whether metformin overcomes crizotinib resistance through the inhibition of the IGF-1R signaling pathway.

In the current study, we found that treatment with metformin lowered the activation of IGF-1R signaling pathway in both parental cells and H2228-CR cells. In addition, metformin reversed the IGF-1-induced drug resistance and decreased IGF-1R signaling activation. Taken together, these findings suggest that metformin reverses crizotinib resistance through the inhibition of the IGF-1R signaling pathway.

### Limitations of the present study

There are several limitations in the present study. First, this is an *in vitro* study, and complete differences exist from identical *in vivo* trials, so more experiments are needed in future studies to investigate the synergistic anti-tumor effect of metformin and crizotinib in xenograft models. Second, the possible molecular mechanisms underlying primary and acquired crizotinib resistance may differ. The two crizotinib-resistant sublines established (H2228-CR and H3122-CR cells) in this study were derived from the parental H2228 and H3122 cell lines by long-term exposure to high concentrations of crizotinib for eight months and can be considered as research on acquired crizotinib resistance. Whether metformin can reverse the primary crizotinib resistance needs to be further confirmed *in vitro* and *in vivo*. Third, we added IGF-1 into the culture medium and observed crizotinib resistance. It would be better to establish cell lines with overexpression of IGF-1 to further investigate the effect of IGF-1-induced crizotinib resistance.

Therapeutic resistance to crizotinib is almost inevitable in patients with ALK rearrangements. Here, we report for the first time that metformin overcomes crizotinib resistance *in vitro* by inhibiting the IGF-1R signaling pathway. Thus, we propose that when combined with crizotinib, metformin can have future, potentially significant clinical utility in the therapy of ALK-positive NSCLC patients.

## MATERIALS AND METHODS

### Cell-lines and reagents

Crizotinib was a gift from Pfizer (PF-02341066) and was prepared in dimethyl sulfoxide (DMSO) to obtain a stock solution of 10 mM. Metformin (Sigma) was dissolved in deionized water and stored at −20°C. The recombinant human IGF-1 was purchased from PeproTech. Crizotinib-sensitive H2228 cells were obtained from the American Type Culture Collection (Manassas, VA, USA) and H3122 cells were obtained from Shanghai Bioleaf Biotech Co., Ltd. (Shanghai, China). Cells were cultured in Roswell Park Memorial Institute 1640 medium (RPMI-1640, HyClone) with Earle's salts, and supplemented with 10% fetal bovine serum (FBS, Gibco), 2 mM L-glutamine solution (Gibco), 100 U/mL penicillin (HyClone) and 100 μg/mL streptomycin sulfate (HyClone) at 37°C, with 5% CO_2_ in the air and 90% humidity.

### Generation of crizotinib-resistant cell lines

Crizotinib-resistant cell lines were generated as previously reported [33]. Generally, parental cells were cultured with increasing concentrations of crizotinib. The doses were augmented in a stepwise pattern when normal cell proliferation resumed. Fresh drug was added every 48–72 hours. Resistant cells that grew in 1 μM crizotinib were derived after approximately eight months of culturing in the continuous presence of the drug.

### MTT assay

The cytotoxic effects of crizotinib plus metformin were determined by the MTT dye reduction assay. A total of 2000 cells were plated in 100 μL culture medium in 96-well microtiter plates. After 24-h incubation, crizotinib and/or 5mM metformin were added to each well as indicated, and the cells were further cultured for 48 h. Then, 10 μL of 5 mg/mL MTT reagent (Sigma) in 100 μL culture medium was added to each well. After 4 h, the medium was removed, and 150 μL of DMSO was admixed to each well to dissolve the formazan crystals. Absorbance was measured at a wavelength of 490 nm by using a ThermoFisher Spectrophotometer1510 (Molecular Devices, Inc.). Cell viability was determined by dividing the absorbance values of the treated cells to that of the untreated cells. The experiments were conducted in triplicates.

### Ki67 incorporation assay

Cell proliferation was also assessed by the Ki67 incorporation assay using a Ki67 labeling and detection kit (Sigma). Briefly, cells were treated with metformin or crizotinib, or both, for 48 h, and then were incubated for 6 h with Ki67 (1:200 dilution) and fixed. Cell nuclei were counterstained with 4′, 6-diamidino-2-phenylindole (DAPI) and then viewed with a live cell station (Delta Vision, API). At least 500 cells from three independent experiments were counted. The data obtained were expressed as the mean value of the percentage of positive cells ± SEM.

### Cell invasion assay

Cell invasion was measured by using 24-well 6.5-mm diameter inserts (8.0 μm pore size, Corning Incorporated). The relative cell invasion index was calculated as reported previously [17]. Triplicate samples of 2 × 10^4^ cells treated as indicated were seeded into the upper well in serum-free medium and incubated with 10% FBS in the lower chambers. Cells were cultured for 48 hours at 37°C with 5% CO_2_. The cells in the upper chamber were then removed with a cotton swab, and those that had migrated into the lower chamber were fixed in 4% paraformaldehyde and stained with 0.1% Crystal Violet. The cells on the bottom side of the filters were counted in 5 random × 100 microscope objective fields.

### Apoptosis assay

Flow cytometric analysis was employed to detect apoptosis by examining altered plasma membrane phospholipid packing by the lipophilic dye Annexin V. Briefly, cells were treated with crizotinib and/or metformin for 48 h, harvested by trypsin, washed twice with PBS, and then resuspended at a density of 1 × 10^7^ cells/mL. Thereafter, 5 μL of Annexin V-FITC and 5 μL of propidium iodide (PI) were added to 100 μL of the cell suspension and incubated for 30 min at room temperature in the dark. Next, labeled cells were analyzed by flow cytometry. All early apoptotic cells (i.e., Annexin V-positive, PI-negative), necrotic/ late apoptotic cells (i.e., double-positive), and living cells (i.e., double-negative) were detected by using a Cytomics FC 500 flow cytometer (Beckman Coulter, Miami, FL, USA).

### Colony-forming assay

The detailed procedures of colony formation assay were described previously [34]. Briefly, 500 cells were resuspended in culture medium and seeded in six-well plates. After 14 days, the cells were washed and fixed with 4% paraformaldehyde. Next, the cells were stained with 0.1% crystal violet. Colonies with a diameter greater than 1 mm were counted. Triplicate samples were used in the experiment.

### Western blot assay

Cells grown and treated as indicated were collected, and the total protein was extracted. The following primary antibodies were used: rabbit monoclonal anti-phosphorylated IGF-1R (Tyr1131), rabbit monoclonal anti-IGF-1R, rabbit monoclonal anti-Akt, rabbit monoclonal anti-phosphorylated Akt (Ser473), rabbit monoclonal anti-phosphorylated mTOR (Ser2448), rabbit monoclonal anti-mTOR, rabbit monoclonal anti-p70s6k, or rabbit monoclonal anti-phosphorylated p70s6k (Thr389), rabbit monoclonal anti-S6, or rabbit monoclonal anti-phosphorylated S6 (Ser240/244), rabbit monoclonal anti-AMPK, or rabbit monoclonal anti-phosphorylated AMPK (Thr172) (all from Cell Signaling Technology, Inc.). Horseradish peroxidase-conjugated goat-anti-rabbit antibody (Thermo Scientific) was used as a secondary antibody. The control for equal protein loading was assessed with an anti-β-actin antibody (Cell Signaling Technology, Inc.).

### Statistical analysis

All data are presented as mean ± standard error of the mean (SEM). Statistical analyses were carried out using the unpaired, two-tailed Student's *t*-test, and statistical significance was assumed at an alpha value of *p* < 0.05.

## SUPPLEMENTARY MATERIALS FIGURES


