# Supplementary material for: Metformin restores crizotinib sensitivity in crizotinib-resistant human lung cancer cells through inhibition of IGF1-R signaling pathway
Source: Oncotarget. 2016 Apr 30;7(23):34442–52. doi: 10.18632/oncotarget.9120 (PMC5085167; doi:10.18632/oncotarget.9120)
Supplement: Supplementary file 1 [file oncotarget-07-34442-s001.pdf]

Metformin restores crizotinib sensitivity in crizotinib-resistant human lung cancer cells through inhibition of IGF1-R signaling pathway

Supplementary Materials

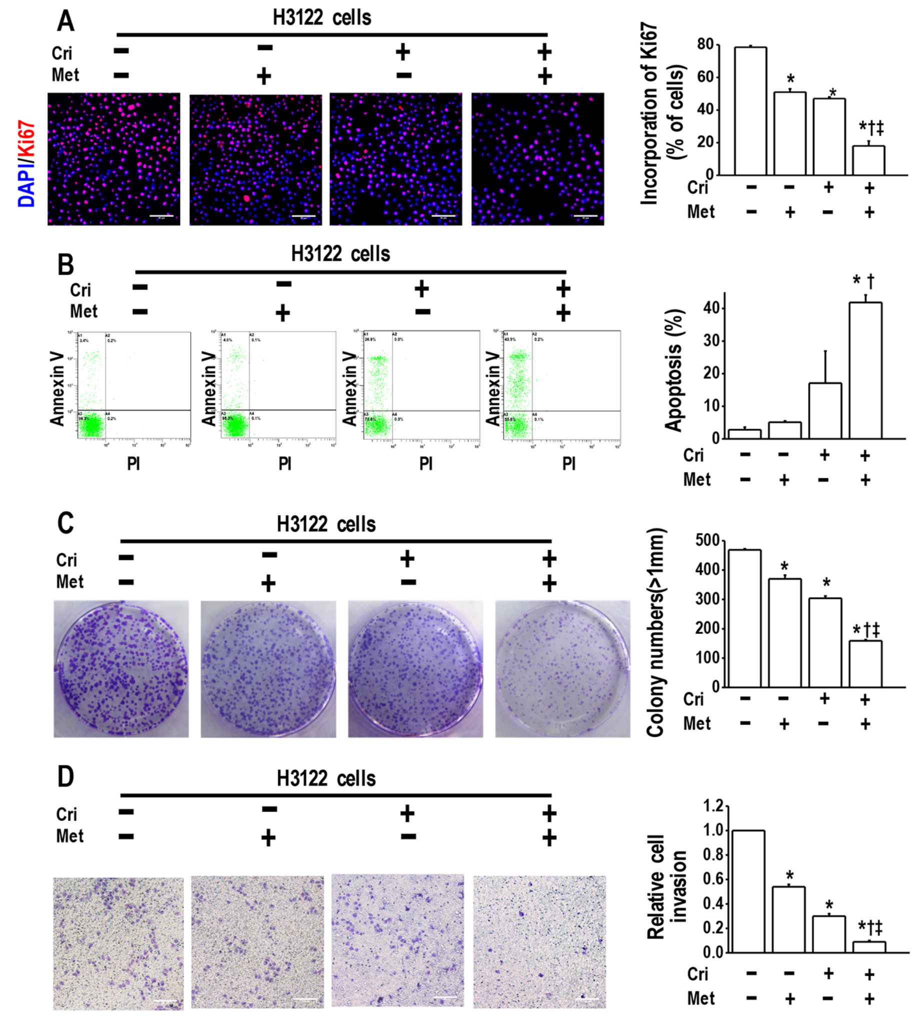

Supplementary Figure S1: Metformin increased crizotinib sensitivity in crizotinib-sensitive H3122 cells. (**A**) Metformin (5 mM) and crizotinib (200 nM) synergistically inhibited the proliferation of H3122 cells, as determined by a Ki67 incorporation assay. *$p < 0.01$ compared with control; †$p < 0.01$ compared with the crizotinib treatment alone; ‡$p < 0.01$ compared with that of metformin treatment alone. Scale bars, 50 µm; (**B**) Metformin (5 mM) in combination with crizotinib (200 nM) significantly enhanced the apoptosis of H3122 cells. The images are representative of three independent experiments. *$p < 0.01$ compared with that of control, †$p < 0.01$ compared with metformin treatment; (**C**) Metformin (5 mM) in combination with crizotinib (200 nM) significantly inhibited colony-forming ability of H3122 cells. Cells were cultured for 14 days and then fixed and stained. Colonies with a diameter greater than 1 mm were counted. *$p < 0.01$ compared with control; †$p < 0.01$ compared with the crizotinib treatment alone; ‡$p < 0.05$ compared with that of metformin treatment alone; (**D**) Metformin (5 mM) and crizotinib (200 nM) synergistically inhibited invasiveness of H3122 cells. Scale bars: 100 µm. *$p < 0.01$ compared with control; †, $p < 0.01$ compared with the crizotinib treatment alone; ‡, $p < 0.05$ compared with that of metformin treatment alone. Met, metformin; Cri, crizotinib.

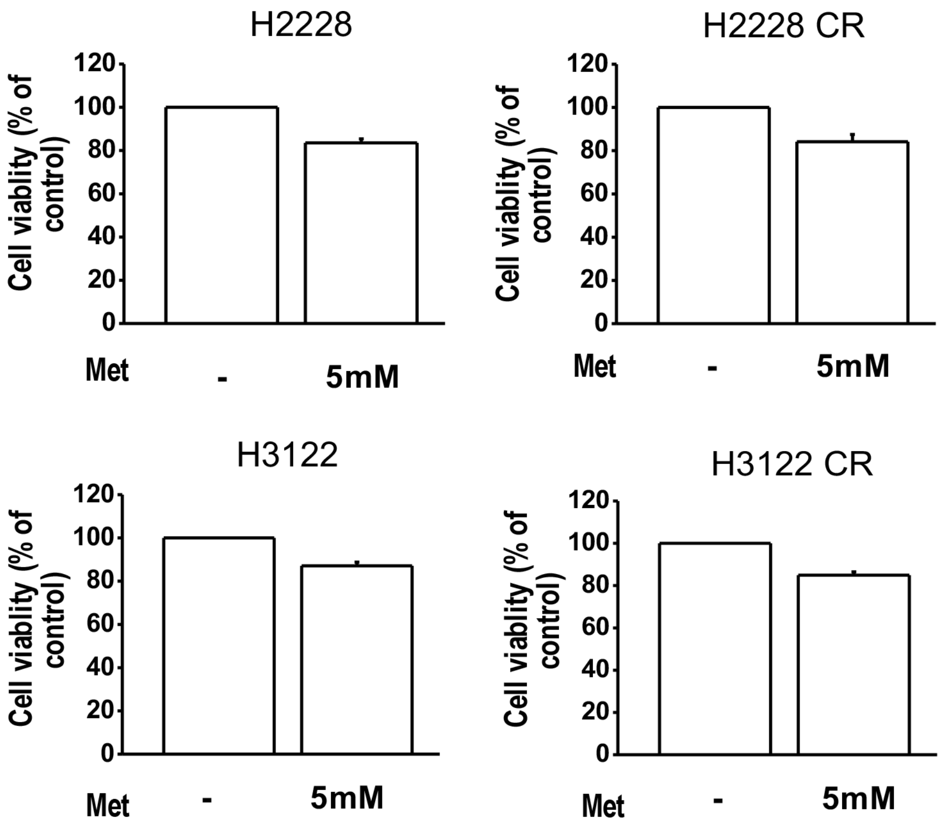

Supplementary Figure S2: Metformin alone slightly decreased cell viability. Metformin of 5 mM slightly decreased cell viability in four types of cells used in the current study.

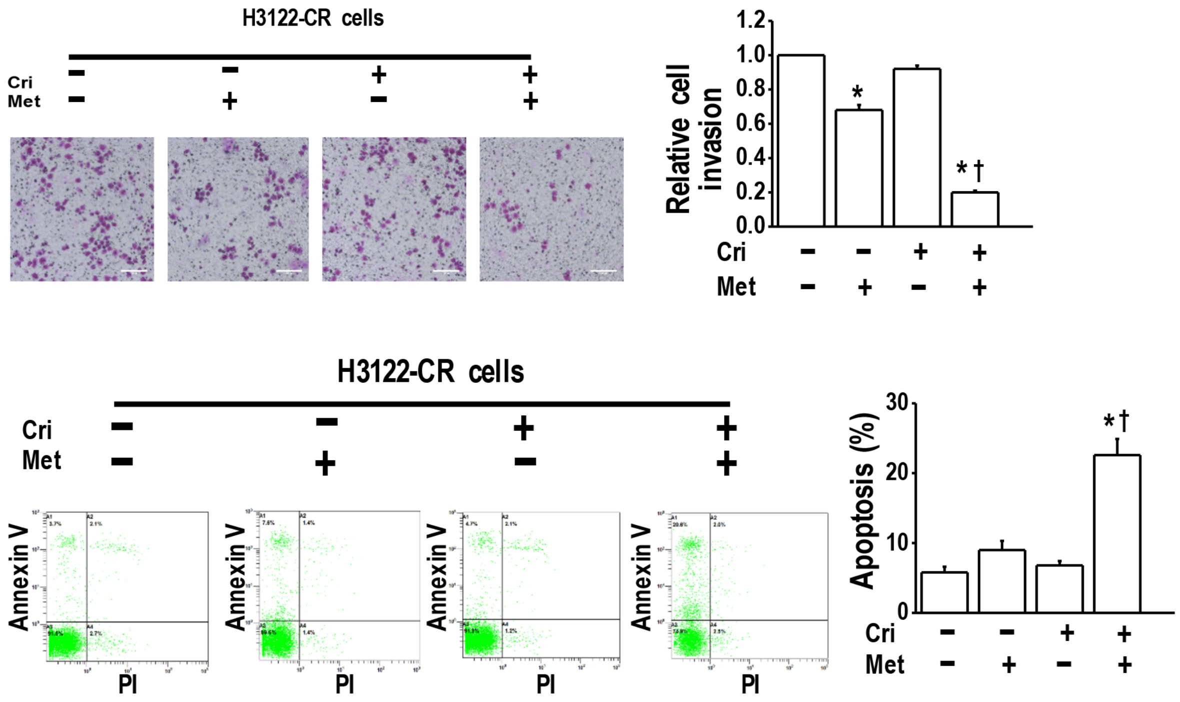

Supplementary Figure S3: Metformin decreased invasion and enhanced apoptosis in H3122-CR cells. (A) Metformin (5 mM) and crizotinib (1 μM) synergistically inhibited invasiveness of H3122-CR cells. Scale bars: 100 μm. *$p < 0.01$ compared with control; †$p < 0.01$ compared with the crizotinib or metformin treatment alone; (B), Metformin (5 mM) in combination with crizotinib (1 μM) significantly enhanced the apoptosis of H3122-CR cells. The images are representative of three independent experiments. *$p < 0.01$ compared with that of control, †$p < 0.01$ compared with metformin treatment; Met, metformin; Cri, crizotinib.
